# Emerging Roles of Type-I Interferons in Neuroinflammation, Neurological Diseases, and Long-Haul COVID

**DOI:** 10.3390/ijms232214394

**Published:** 2022-11-19

**Authors:** Ping-Heng Tan, Jasmine Ji, Chung-Hsi Hsing, Radika Tan, Ru-Rong Ji

**Affiliations:** 1 Department of Anesthesiology, Chi Mei Medical Center, Tainan 701, Taiwan; 2 Neuroscience Department, Wellesley College, Wellesley, MA 02482, USA; 3 Kaohsiung American School, Kaohsiung 81354, Taiwan; 4 Center for Translational Pain Medicine, Department of Anesthesiology, Duke University Medical Center, Durham, NC 27710, USA; 5 Departments of Cell Biology and Neurobiology, Duke University Medical Center, Durham, NC 27710, USA

**Keywords:** neuroinflammation, neurological disease, microglia, astrocytes, IFN-α, IFN-β, primary sensory neurons, pain, spinal cord, long-haul COVID

## Abstract

Interferons (IFNs) are pleiotropic cytokines originally identified for their antiviral activity. IFN-α and IFN-β are both type I IFNs that have been used to treat neurological diseases such as multiple sclerosis. Microglia, astrocytes, as well as neurons in the central and peripheral nervous systems, including spinal cord neurons and dorsal root ganglion neurons, express type I IFN receptors (IFNARs). Type I IFNs play an active role in regulating cognition, aging, depression, and neurodegenerative diseases. Notably, by suppressing neuronal activity and synaptic transmission, IFN-α and IFN-β produced potent analgesia. In this article, we discuss the role of type I IFNs in cognition, neurodegenerative diseases, and pain with a focus on neuroinflammation and neuro-glial interactions and their effects on cognition, neurodegenerative diseases, and pain. The role of type I IFNs in long-haul COVID-associated neurological disorders is also discussed. Insights into type I IFN signaling in neurons and non-neuronal cells will improve our treatments of neurological disorders in various disease conditions.

## 1. Introduction

Interferons (IFNs) were first found in 1957 [1] and were found to be able to “interfere” with viruses [2]. In addition to antiviral effects, IFNs also could affect the function of the immune system, endocrine system, and nervous system, especially the central nervous system (CNS). As a whole, the IFN family can be divided into three subfamilies: the type I IFNs (IFN-Is), the type II IFN (which contains IFN-γ), and the type III IFNs (which contain IFN-λ1-3) [1,3]. IFN-Is include IFN-α (13 homologous human and 14 homologous mouse subtypes), IFN-β, IFN-δ, IFN-ε, IFN-κ, IFN-τ, and IFN-ω1–3. IFN-α, IFN-β, IFN-ε, IFN-τ, IFN-κ, and IFN-ω are human IFNs [4]. This review article will focus on IFN-α and IFN-β. IFN-I family members are pleiotropic cytokines, and they are potent immunomodulatory factors that bridge the innate and adaptive immune responses and act as antimicrobial, antitumor, and pain mediators in the host [4,5,6].

**Figure 1:**
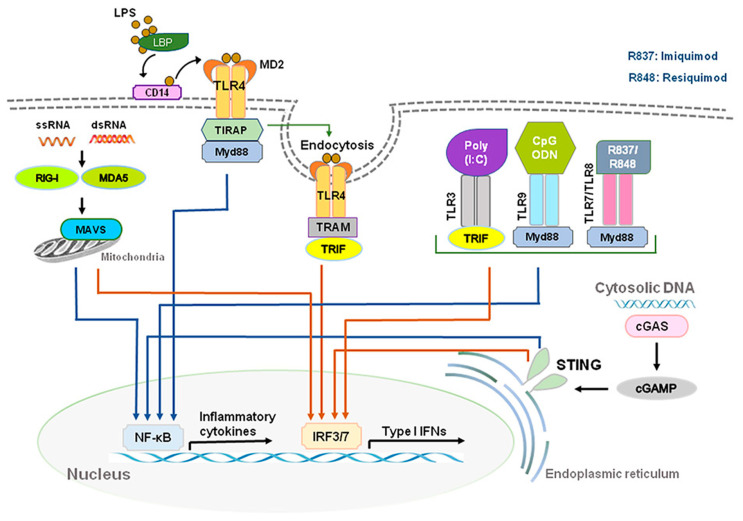
Production of type I IFNs. Type I IFNs are induced by activation of toll-like receptors (TLRs) and stimulator of interferon genes (STING). Abbreviations: CD14, cluster of differentiation 14; LBP, LPS-binding protein; LPS, lipopolysaccharide; MD2, myeloid differentiation factor 2; poly(I:C), polyinosine-deoxycytidylic acid; poly(I:C), polyinosine-deoxycytidylic acid; IRF3/7, interferon regulatory factor 3/7; MYD88, myeloid differentiation primary response 88; NF-κB, nuclear factor kappa-light-chain-enhancer of activated B cells; STING, stimulator of interferon genes; TRIF, TIR-domain-containing adapter-inducing IFN-β.

During viral or bacterial infection and tissue injury, cytokines and chemokines, including IFN-Is, are released through activation of pattern recognition receptors (PRRs) such as toll-like receptors (TLRs) that sense pathogen/damage-associated molecular patterns (PAMPs/DAMPs) through neuro-immune interactions [7,8]. TLR 2, 4, and 5, localized on outer membrane, respond primarily to bacterial surface associated PAMPs. TLR4 can also be found to a lesser extent in endosomes. TLR3 and TLR7/8, localized on endosomes, mainly respond to nucleic-acid-based PAMPs from viruses and bacteria such as double-stranded RNAs (dsRNA) and single-stranded RNAs (ssRNA), respectively [9,10]. TLR3 recognizes viral dsRNA and its synthetic analog, polyinosine-deoxycytidylic acid (poly(I:C)). Imiquimod and Resiquimod are imidazoquinoline-like molecules that have been identified as TLR7/8 agonists. TLR9 is localized on endosomes and senses double-stranded DNA (dsDNA) and CpG unmethylated DNA. IFN-I is induced by TLR4 upon recognition of lipopolysaccharides (LPS) and viral proteins from Gram-negative bacteria [7]. Activation of TLRs can produce large amounts of IFN-I and induce subsequent specific intracellular signaling pathways after sensing PAMPs and DAMPs. The ligand binding to the TLR initiates recruitment to the receptor of adaptor proteins TRIF (for TLR3) and MyD88 (for TLR7/8/9). In turn, this initiates a cytoplasmic signaling cascade that leads to the phosphorylation of IFN regulatory factors (IRFs) 3 and 7 by TANK binding kinase (TBK) and the activation of nuclear factor kappa-light-chain-enhancer of activated B cells (NFκB). Transcription of IFN-I genes is then mediated by IRFs binding to promoter/enhancer regions [11] (Figure 1).

DExD/H-box RNA helicases and stimulator of interferon genes (STING) situated in the cytoplasm are also capable of detecting microbial RNA and DNA and producing IFN-I, in addition to TLRs [6]. The family includes three type of receptors, such as the retinoic acid inducible gene-1(RIG-I)-like helicases (RLHs), RIG-I, and melanoma differentiation-associated protein 5 (MDA5) [2,11]. STING is a protein with four putative transmembrane domains and resides in the endoplasmic reticulum (ER) and activates intracellular DNA-mediated production of IFN-I [9,12,13] (Figure 1). STING can be activated after binding cyclic dinucleotides (CDNs) derived from virus or intracellular DNA and activates the kinase TBK1 to induce phosphorylation of STING. Phosphorylated STING is coupled with TBK1 and recruits IRF3 to enter the nucleus, activating the production of IFN-I and promoting the eradication of pathogens mediated by immune cells [12,14]. Different types of cells could produce IFN-α and IFN-β, including macrophages, natural killer (NK) cells, fibroblasts, B cells, T cells, and osteoblasts. IFN-α and IFN-β can exert anti-viral and anti-tumor effects by stimulating NK cells and macrophages. During virus infection or stimulation with DNAs/RNAs, IFN-I is mainly produced and secreted by the plasmacytoid dendritic cells (pDC) [15,16,17]. 

## 2. Intracellular Signaling of IFN-I 

**Figure 2:**
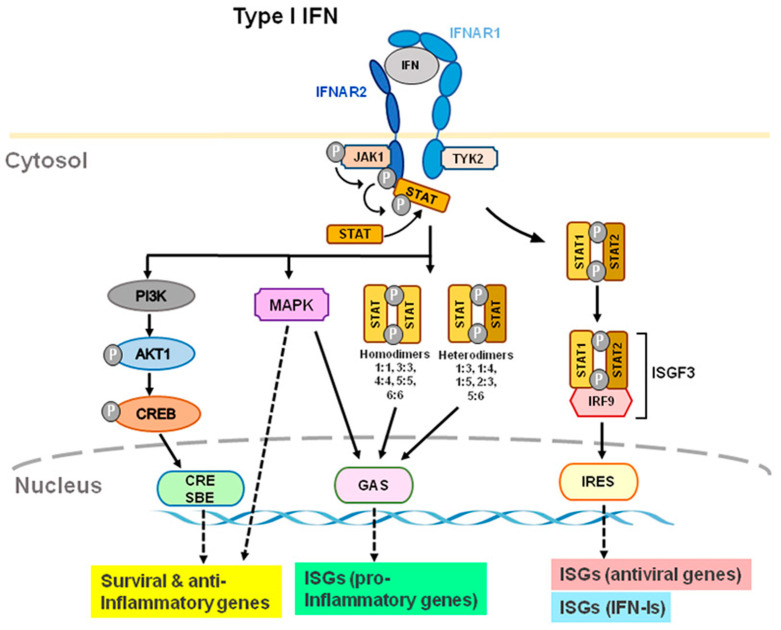
Type I IFN-induced interferon receptor (IFNAR) signaling and gene expression. IFN-α and IFN-β transmit signals via IFNAR1 and IFNAR2 subunits. TYK2 and JAK1 are distinct components of IFNAR1 and IFNAR2. Through ISRE, GAS, and CRE/SBE, TYK2 and JAK1 activation lead to activation of STAT1/2, STAT homo- or heterodimers, PI3K, and MAPK, which activates transcription of ISGs, including antiviral, type-I IFN, and pro-inflammatory genes. Furthermore, TYK2 may form associations with membrane proteins, such as ion channels, which modulate the activity of cells rapidly. Abbreviations: JAK1, Janus kinase 1; TYK2, tyrosine kinase 2; STAT, signal transducer and activator of transcription; MAPK, mitogen-activated protein kinase; PI3K, phosphoinositide 3-kinase; CRE, cyclic AMP response element; SBE, SMAD binding elements; GAS, IFN-γ-activated sites; ISRE, IFN-stimulated response elements; ISG, IFN-stimulated genes.

IFN-Is bind to heterodimer interferon receptors (IFNAR1 and IFNAR2) [4,18,19,20,21] and subsequently recruit Janus family kinase1 (Jak1) and tyrosine kinase 2 (Tyk2) to phosphorylate and activate IFNAR1 and IFNAR2. JAKs are composed of the four family members of JAK1, JAK2, JAK3, and tyrosine kinase 2 (TYK2). IFNAR1 is coupled with TYK2, whereas IFNAR2 is coupled with JAK1 (Figure 2). In humans, the genes that code for IFN-Is are located on chromosome 9; these genes are located on chromosome 4 in mice [22]. IFNAR1 signaling is dependent on TYK2; IFNARII signaling is dependent on JAK1 (Figure 2). After receptor subunits rearrange and dimerize in response to ligand, these receptor-associated JAKs autophosphorylate to activate STAT (signal transducer and activator of transcription) [23]. IFNARs are activated and subsequently phosphorylate effector proteins of the signal transducers and activators of transcription (STAT) family [4]. Phosphorylated STAT1 and STAT2 couple with IRF9 to form the transcription factor ISGF3 and translocate to the nucleus to stimulate the transcription of IFN-stimulated genes (ISG), including antiviral genes and type I IFNs themselves [17,18] (Figure 2). Antiviral genes include ISG 15, 2′-5′ oligoadenylate synthetase (OAS), ribonuclease L (RNase L), orthomyxovirus resistance gene (Mx), and viperin, etc. Many of the biological effects of IFN-Is appear to be mediated by the activation of JAK-STAT signaling pathways. Activation of IFNAR1/2 also results in non-canonical signaling such as the activation of PI3 kinase (PI3K) and mitogen-activated protein kinase (MAPK) signaling pathways [6] in addition to canonical signaling pathway (Figure 2).

Upon activating IFNRs and downstream signaling, IFN-I could induce multiple antiviral genes to inhibit virus replication in infected cells and prevent infection of nearby cells [3]. The suppressor of cytokine signaling (SOCS)1 is a potent inhibitor of JAK2 and could reduce IFN-Is response. Production of suppressor of SOCS1 expression is maintained by IFN-β or high amounts of IFNɑ2 [24]. SOCS-1 also enhances immunological actions of IFN-γ but inhibits the adverse effect of unregulated IFN-γ responses by inhibiting STAT1ɑ and decreasing the duration of IFN-γ signaling. By either binding directly to receptors or inhibiting JAK1, JAK2, and TYK2 directly, SOCS3 inhibits the receptor signaling but not JAK3 signaling. In comparison to SOCS1, SOCS3 is less potent than SOCS1 in suppressing IFN-γ signaling. By suppressing STAT3 signaling, SOCS3 also inhibits Th17 differentiation. Through IL-6 signaling, SOCS5 negatively regulates STAT6, an essential signaling molecule for IL-4, which inhibits Th2 differentiation [20].

Many types of viral infections, including hepatitis C and hepatitis B, have been successfully treated with IFN-Is. Multisystem autoimmune diseases, such as multiple sclerosis, are also treated with IFN-Is [25]. IFN-Is also were used to treat cancer through directly activating cytotoxic T lymphocytes, NK cell activation, induction of tumor cell death, and inhibition of angiogenesis. Treatment for melanoma with IFN-Is is reported to effectively improve disease-free survival [26]. IFN-Is can interact with host cells to induce protective immunity; e.g., IFN-I can enhance antibody production by dendritic cells. IFN-Is were also immunosuppressants and anti-inflammatory mediators by induction of programmed cell death-ligand 1 (PD-L1) [27] and anti-inflammatory cytokines (e.g., IL-10) [14] as well as blocking the expression of pro-inflammatory mediators, such as matrix metalloproteinase 9 (MMP-9), ICAM-1, VCAM-1, and tumor necrosis factor-α (TNF-α) [28,29].

IFN-Is were extensively used to treat chronic inflammatory diseases, including autoimmune disorders such as MS, chronic viral infections, and malignant tumors. However, IFN-I-based treatments also produce significant adverse effects, including neurological and neuropsychiatric disorders and various systemic autoimmune diseases, such as rheumatoid arthritis, Aicardi–Goutières syndrome (AGS), systemic lupus erythematosus, Sjogren’s syndrome, and systemic sclerosis etc. [30]. Additionally, inhibition of B-cell activity or production of immunosuppressive molecules (e.g., IL-10) were reported by high concentrations of IFN-I treatment of long-term viral infections [14]. Therefore, IFN-I has pleiotropic effects, mobilizing immune cells to destroy viruses and bacteria on one hand and inducing neuroinflammation on the other. These complications or diseases have been termed “type I interferonopathies”. The following will discuss in more detail the beneficial and detrimental effects of type-I IFNs in the nervous system.

## 3. IFN-Is in Neuroinflammation, Pain, and Neurological Diseases 

IFN-Is are important to maintain CNS homeostasis. For example, IFN-β plays a protective role by promoting the secretion of nerve growth factor [31]. Furthermore, knockout mice lacking IFN-β show defects in neuronal survival, neurite outgrowth, and branching and developed Parkinson’s disease (PD)-like neurodegeneration [32]. IFN-Is possess potent anti-inflammatory properties in the immune system and the CNS [28,29,33]. For example, IFN-β reduces the trafficking of inflammatory cells across the BBB, increases CNS levels of the anti-inflammatory cytokine IL-10, and inhibits the expression of the pro-inflammatory cytokine IL-17 as well as increases the number of natural killer (NK) cells and produces anti-inflammatory substances in the peripheral blood [33,34]. The plasmacytoid dendritic cells (pDCs) were the major IFN-I-producing cells in humans and mice [15,17]. However, no pDCs and no specialized IFN-I-producing cells equivalent to pDCs were detected in the brain parenchyma [17,35]. Therefore, sensing pathogens and the induction of the innate immune response and subsequent production of IFN-I in the brain depend on the resident cells of the CNS, such as glial cells (e.g., microglia and astrocytes) and neurons [36,37,38]. 

Increasing evidence suggests that IFN-Is play important roles in regulating encephalitis, depression, cognition/aging, and neurological diseases such as Alzheimer’s disease (AD) and multiple sclerosis (MS), PD (Table 1). Neuroinflammation in the CNS has been strongly implicated in various neurological diseases such as stroke, traumatic brain injury, spinal cord injury, and chronic pain as well as neurodegenerative diseases such as AD, MS, and PD [39,40,41,42,43,44]. Neuroinflammation is characterized by infiltration of immune cells, which are associated with the disruption of the blood–brain barrier (BBB) [43]; the activation of glial cells, such as microglia and astrocytes; and the production of inflammatory mediators, such as pro-inflammatory cytokines and chemokines as well as proteases (e.g., MMP-9) [41,45,46]. IFN-Is contribute to various neurological and neurodegenerative conditions by regulating neuroinflammation.

A major role is played by IFN-Is in the control of encephalitis. It is not clear what their role is during viral encephalitis, but they play an important role during sterile neuroinflammation, during which microglia play a pathological role. Many viruses enter the CNS through the olfactory bulb [47]. The olfactory bulb accumulates microglia and monocytes after intranasal injection of vesicular stomatitis virus. In encephalitis, depletion of microglia promotes virus spread, which results in an increased mortality rate [47]. The activity of IFN-I IFNs in astrocytes was also reported to protect against viral encephalomyelitis and to be involved in the expression of IFN-gamma [71]. Furthermore, the IFN-I receptor signaling on neurons plays a crucial role in the activation of myeloid cells. It is essential that neurons, astrocytes, and microglia communicate in the infected CNS to prevent encephalitis from becoming fatal [47]. There is an increased secretion of IFN-α and IFN-β in viral encephalitis [35] as well as by microglia and astrocytes in HIV-1-associated encephalitis [37]. AGS is characterized by chronically elevated IFN-α production in the central nervous system. In the absence of congenital viral infection, AGS often mimics congenital viral infections. However, molecular genetic studies have identified mutations in six genes that can cause the condition, probably through dysregulated nucleic acid metabolism and activation of the innate immune system that leads to increased IFN-α production intrathecal. IFN-α was mainly secreted from astrocytes in AGS [72]. Additionally, in vivo infection of mice with Theiler’s virus or La Crosse virus or neurons differentiated from the human NTera-2 cell line can produce IFN-β [73]. Furthermore, Delhaye et al. (2006) also reported that macrophages and microglia, ependymal cells, along with neurons, produce the IFNs in mice infected with Theiler’s and La Crosse viruses. However, IFN-I production in neurons appears to be limited since fewer than 3% of infected neurons expressed IFN [35]. The expression of IFN receptors in different cell types, including macrophages, monocytes, T lymphocytes, glia, and neurons, is noteworthy [1,74]. 

Depression is a major side effect of IFN-I immunotherapy. IFN-α is used to treat cancers (including melanoma and lymphoma) [75] and hepatitis C virus (HCV). However, patients who undergo immunotherapy with IFN-α often experience depressive symptoms, with half of patients developing some disturbance in concentration, drive, or mood [48]. Studies have demonstrated that IFN-α can induce depressive symptoms or exacerbate pre-existing depressive symptoms when taken 1–3 times per week for 1–3 months [49]. Patients who developed IFN-α-induced depression are also at higher risk of recurrent depression [76]. IFN-α may drive the development of depressive symptoms via different mechanisms. IFN-α can induce indoleamine-2,3 dioxygenase-1, which activates the kynurenine pathway, leading to dysregulation of serotonergic and dopaminergic metabolism that has been linked to major depression [77]. IFN-α can increase the IL-6 level, which contributes to the development of depressive symptoms [49]. IFN-α can also cause immune responses and dysregulations in the hypothalamo-pituitary-adrenal (HPA) axis, which was seen in patients with major depression [48]. Intracerebroventricular administration of IFN-α in rhesus monkeys confirmed that central IFN-α administration could produce depressive behavior [50]. Because immunotherapy with IFN-α can induce depressive symptoms, more specifically, targeted drugs have begun to replace IFN-α in the treatment of cancers and viruses including HCV [48]. However, IFN-α’s ability to induce depressive symptoms is worth studying. Murine models of HCV treated with IFN-α and polyinosinic acid, an agonist of TLR3 that can mimic HCV-double-strand RNA, intriguingly revealed that only the delivery of both IFN-α and polyinosinic acid resulted in depressive symptoms, suggesting the necessity of a pathological condition for IFN-ɑ to produce depressive symptoms [53]. IFN-ɑ-induced depression is also a promising model for major depression although IFN-α causes more somatic symptoms other than mood and cognition [78]. Currently, the most effective treatments for IFN-α-induced depression are selective serotonin reuptake inhibitors (SSRIs), which can be supplemented with hydroxytryptophan and tryptophan [79]. 

The prolonged exposure to IFN-Is during immunotherapies has been shown to induce cognitive impairment and even delirium in both mice and patients [51,52]. Type I IFNs have been implicated in aging, which is accompanied by cognitive decline (Table 1). Choroid plexus produces cerebrospinal fluid (CSF) and separates CSF from the blood, and recent studies show that the choroid plexus exhibits type I IFN-dependent gene expression profile in both aged mice and humans. Brain-derived signals in the CSF were able to induce a type I IFN response in mice [80]. Such IFN-I response is not only a phenotype of aging but also contributes to the senescence-associated secretory phenotype [81]. Meanwhile, cognitive function was restored to some extent in mice when IFN-I signaling was blocked using intracerebroventricular administration of IFN-I receptor-neutralizing antibody to the CSF of the aged mice, implicating IFN-I in cognitive decline with aging [80,82]. Mechanistically, IFN-α can disrupt endogenous neurogenesis, neurite outgrowth, neuronal survival, and neurotrophic signaling in the hippocampus of murine models [53]. Importantly, IFN-α and IFN-β can directly act on neurons to modulate neuronal activities as neuromodulators. For example, IFN-α inhibits long-term potentiation (LTP) in hippocampal neurons, a cellar substrate of learning and memory [83]. IFN-β was shown to reduce the amplitude of striatal excitatory post-synaptic currents, indicating an inhibitory effect on glutamate neurotransmission, especially the NMDA component [84].

AD, a condition seen in many aging people, is a neurodegenerative disorder associated with reductions in memory functioning and cognition. IFN-I s may also play a role in the development of AD. In human brains with AD, IFN-α was strongly upregulated in microglia [56,85]. One study using PDGFB-APPSwInd (J20) transgenic mouse model of AD found an overexpression of IFN-β in mice of all ages accompanied by a high expression of IFN-γ. The overexpression of IFN-β was associated with memory impairment, increased glial activation, and the overexpression of IFN-I-induced genes, including *Ifi27I2a*, *Oas1*, *Irf7*, and *Cxcl10* in the dorsal hippocampus following a three-month period [55]. In a separate study, an IFN-stimulated gene signature was found in the brains of various murine AD models. Administering recombinant IFN-β led to microglial activation and elimination of C3-dependent synapse, while blocking IFNAR decreased microgliosis and synapse loss in these models. These findings indicate that IFN-β plays a significant role in elevating AD-associated neuroinflammation [40]. Upregulation of IFN-I contributes to the pathogenesis of AD in mice [86]. In contrast, IFN-I signaling through STAT1 may also reduce the activity of the Nlrp3 inflammasome, which plays an active role in neuroinflammation and AD [87].

IFN-I s may also play a role in PD [58,59]. Increased IFN-I signaling was found in both the MPTP mouse model as well as the postmortem human Parkinson’s disease samples [60]. In the 1-methyl-4-phenyl-1, 2, 3, 6-tetrahydropyridine (MPTP) model, mice without IFNAR^(−/−)^ showed decreased IFN-I signaling and a weaker pro-inflammatory response in addition to a reduction in the loss of dopaminergic neurons. Reduced neuroinflammation and dopaminergic cell death in mice treated with monoclonal IFNAR1 (MAR-1) antibody further confirms the neuroprotective potential of targeting the IFN-I pathway [59,60]. Furthermore, mice lacking STING, a key inducer of type I IFNs, seems to be protected against the PD-associated neuroinflammation [58]. Thus, IFN-I is a key modulator of neuroinflammatory response that plays a significant role in the pathogenesis of PD [59].

IFN has stood out as the most promising treatment for MS. In MS, an autoimmune disease, the body’s immune cells attack its myelin, resulting in breaks in neural circuits that prevent the proper functioning of the CNS. Administering IFN-β effectively decreases both the number and severity of MS attacks, slowing the progression of the disease [25]. IFN-β prevents the myelin-damaging products from being produced, suggesting it may have regulatory roles in the brain, endocrine system, and immune system [25]. IFN-β decreases serum levels of cytokines such as TNF-α [88] as well as MMP-9 activity and the ratio of MMP-9 and TIMP-1 (an endogenous inhibitor of MMP-9) in blood samples in multiple sclerosis [61,62]. Notably, induction of IFN-I is protective against and is effective against experimental autoimmune encephalomyelitis (EAE), an animal model for MS [89]. Notably, there are also MS conditions that are resistant to IFN-β treatment [90]. Type-I IFN signaling can also be detrimental. Experimental autoimmune encephalomyelitis (EAE) induced by injection of antibodies against myelin oligodendrocyte glycoprotein (MOG) is reduced in knockout mice lacking IFNAR [63]. 

A large body of preclinical literature has demonstrated a key role of neuroinflammation characterized by glial activation and production of proinflammatory mediators [91,92,93]. Neuroinflammation also occurs in clinical chronic pain states, adding further weight to the preclinical studies. The analgesic effects of IFN-α in the CNS have been reported to be through activating opioid receptors (Table 1). IFN-α was initially shown to produce anti-nociceptive effect through activating mu- but not delta- and kappa-opioid receptors in the nucleus submedius [94]. It was also shown that IFN-α produces an antinociceptive effect by sharing similar pharmacological properties with β-endorphin [64]. Furthermore, IFN-α inhibited the binding of [3H]-naloxone to opioid receptor in vivo, demonstrating competition between IFN-α and naloxone on membrane binding sites [95].

**Figure 3:**
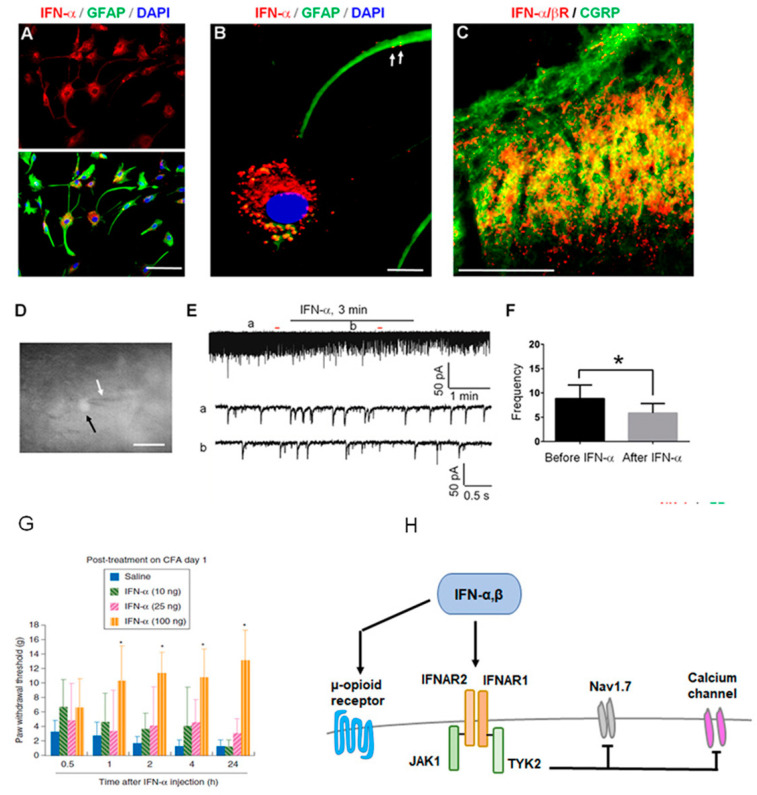
Expression of interferon (IFN)-α, interferon receptor (IFNAR) and IFNAR signaling in nociceptive sensory neurons and their central terminals producing antinociceptive effects. (**A**,**B**) IFN-α expression in cultured astrocytes (**A**). (**B**) Triple staining of IFN-α, GFAP, and nucleus marker DAPI in astrocytes. Scales: 50 μm in A and 10 μm in B. Arrows indicate IFN-α-labeled vesicles in remote astrocyte processes. (**C**) IFNAR (IFN-α/βR) is colocalized with CGRP in the superficial dorsal horn. Scale: 100 μm. (**D**–**F**) IFN-α inhibits synaptic transmission in spinal cord slices. (**D**) The recorded neuron (somatostatin-positive, shown by white arrow) with an electrode (black arrow). (**E**) Inhibition of spontaneous excitatory postsynaptic currents (sEPSCs) in lamina II neurons by IFN-α was shown by patch clamp (25 ng/mL, 2 min). a and b indicate traces before and after the treatment. (**F**) Frequency of sEPSCs, expressed as ratio of baseline. Seven out of nine recorded neurons respond to IFN-α. * *p* < 0.05, *n* = 7 neurons. (**G**) Intrathecal injection of IFN-α produces dose-dependent inhibition of inflammatory pain in rats. IFN-α was given one day after inflammation by complete Freund’s adjuvant (CFA). (**H**) Type I IFN (IFN-I) activates IFNAR1, resulting in subsequent TYK2 activation and rapid antinociception via inhibition of Na^+^ and Ca^2+^ channels and suppression of action potential firing in nociceptors. In addition, IFN-β may also produce antinociception via interaction with mu-opioid receptors. A–F are reproduced “Reprinted/adapted with permission from Ref. [66]. 2016, Tan PH”. “G is reproduced from Reprinted/adapted with permission from Ref. [65]. 2012, Tan PH”.

Interestingly, IFN-α was markedly upregulated after intrathecal administration of short (<21 bp) ds RNA at high doses (>10 ug) [65]. DsRNAs with long lengths (>30 bp) induce an IFN response by activating protein kinase R in mammalian cells as a non-specific viral defense [96]. Short dsRNAs and short hairpin RNAs are able to induce IFN responses in vitro [97]. DsRNAs may also activate IFN via TLR7 and TLR8, which are recognized by dsRNAs in the endosome, apart from responses mediated by protein kinase R [98]. The reversal of IFN-induced analgesia by naloxone shows that the antinociceptive effect of IFN-α acts on the opioid receptor (Figure 3). Before this report, no report had demonstrated that short dsRNAs produce analgesic effects through the IFN-α response in the spinal cord. This is the first report demonstrating the IFN-α-mediated analgesic effects of dsRNAs in the spinal cord. Thus, these findings indicate that caution must be taken when designing dsRNAs for target validation in pain research. 

**Figure 4:**
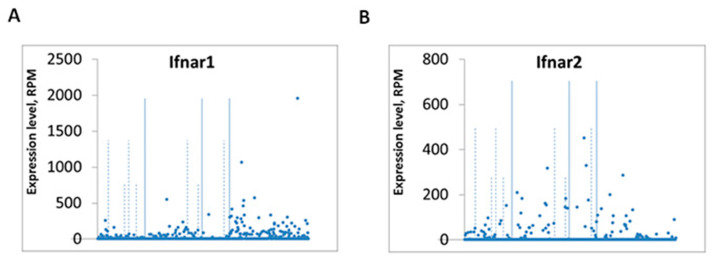
Single-cell RNAseq showing the expression of *Ifnar1* (**A**) and *Ifnar2*1 (**B**) in different populations of mouse DRG neurons. Search engine of scatter plots of expression, reads-per-million (RPM), for any gene in 622 neurons assigned to individual populations. Values are grouped along the horizontal axis according to identified populations. Solid vertical lines separate major sensory neuron types in the order: NF (neurofilament), NP (non-peptidergic), PEP (peptidergic), TH (tyrosine hydroxylase) populations. Dashed vertical lines separate major populations into further subtypes, for example, NF1 to NF5 for NF major type. Vertical axis shows normalized expression level in RPM (reads per million) for individual cells. Each dot represents one neuron. Plotted from the database of Usoskin et al., 2015 [99].

Single-cell RNA sequence (sc-RNAseq) in dissociated mouse DRG neurons revealed broad expression of *Ifnar1* and *Ifnar2* in different types of primary sensory neurons, including peptidergic, non-peptidergic, and myelinated neurons [99] (Figure 4). Furthermore, sc-RNAseq shows *Ifnar1* and *Ifnar2* expression in mouse trigeminal neurons. Importantly, sc-RNAseq shows *IFNAR1* and *IFNAR2* expression in human trigeminal neurons [100]. In situ hybridization revealed *Ifnar1* mRNA expression in primary sensory neurons of dorsal root ganglion (DRG) [99]. In the spinal cord, IFN-α is mainly expressed by astrocytes (Figure 3A,B). Furthermore, the IFN-α/β receptor can be expressed by mouse DRG neurons as well as in primary afferent terminals in the superficial dorsal horn, which co-expresses the neuropeptide CGRP [66] (Figure 3C). By perfusing spinal cord slices with IFN-α, administration of IFN-α could suppress the frequency of spontaneous excitatory postsynaptic current (sEPSCs), which means suppression of excitatory synaptic transmission on somatostatin-positive neuron (Figure 3D–F). The study also showed that IFN-α treatment in spinal cord slices suppressed nociceptive transmission in the spinal cord pain circuit by blocking the capsaicin-induced internalization of NK-1 and phosphorylation of extracellular signal-regulated kinase (ERK) in the superficial dorsal horn neurons. When naive rats were intrathecally injected with IFN-α, their pain thresholds increased in both naïve and inflamed conditions (Figure 3G). By contrast, administering a neutralizing antibody to remove endogenous IFN-α resulted in hyperalgesia [5,65]. From this study, it appears that there may be a form of neuronal–glial interaction by which IFN-α can inhibit nociceptive transmission in the spinal cord. 

A recent study reported that STING-mediated antinociception is governed by IFN-Is, which rapidly suppress excitability of mouse, monkey, and human nociceptors [5,101] (Figure 3H). Knockout mice lacking STING or IFN-I signaling exhibited hypersensitivity to nociceptive stimuli and heightened nociceptor excitability. Furthermore, Ifnar1−/− mice and mice lacking Ifnar1 selectively in sensory neurons exhibited robust hypersensitivity to mechanical and cold stimuli. IFN-I signaling can directly suppress nociceptor excitability via suppression of sodium and calcium channel activity. Additionally, Stokes et al. found intrathecal IFNβ (100 ng/5μL) could relieve allodynia induced by intrathecal TLR2 or TLR4 ligands. Intrathecal TLR3-L (Poly(I:C)) produced a prolonged allodynia than intrathecal TLR4-L (LPS) in the Ifnar1−/− mice [67]. This implies that the rapid resolution of allodynia may require IFN-I signaling. 

Study also reported that intrathecal administration of IFNβ of 1000 u, 5000 u, and 10,000 u produces significant, transient, and dose-dependent attenuation of mechanical allodynia for neuropathic pain. This analgesic effect is mediated by induction of the ubiquitin-like protein ISG15. Activation of ISG15 could further inhibited MAPK signaling of pERK, pJNK, and pP38. ISG15 was found to be colocalized mostly with neurons and astrocytes and some microglial cells in the superficial layers of the dorsal horn. These findings first highlight the immunomodulation for IFNβ, ISG15, and MAPK signaling in treatment of neuropathic pain [68]. Intrathecal IFN-β is also effective in attenuating arthritic pain [69]. While intrathecal IFN-β alone produced transient pain relief, a combination of intrathecal IFN-β with anti-TNF-a antibody could attenuate arthritic pain for several weeks. This long-term pain relief was significantly regulated by downstream effectors activated through interferon inducible factors including but not limited to IL-10 [69].

It was also found intraplantar administration of either IFN-α (300 U/25 uL) or IFN-β (300 U/25 uL) but not vehicle (saline) produced a rapid mechanical hypersensitivity lasting for at least 3 d in von Frey test in mice. Furthermore, exposure of DRG neurons to IFN-α (300 U/mL) resulted in neuronal excitability using patch-clamp electrophysiology. Type I interferons induce MNK-mediated eIF4E phosphorylation in DRG neurons to promote persistent pain hypersensitivity. Formalin-evoked nociceptive behavior was also potentiated in mice treated with IFN-α administration for 8 days (8000 IU/g/day). Formalin-evoked inflammatory nociceptive behavior was not altered by single injection of IFN-α [70]. Notably, this is a dose much higher than that producing analgesic effects [65,68]. Furthermore, because peripheral IFN-β cannot directly penetrate the BBB to reach the CNS [78], pronociceptive effects may be mediated by peripheral and local effects [78]. It is possible that the mechanisms of IFN-α or IFN-β on nociceptor central terminals in the CNS vs. nociceptor peripheral terminals in the skin are different.

## 4. Role of IFN-Is in Long-Haul COVID Syndrome 

**Figure 5:**
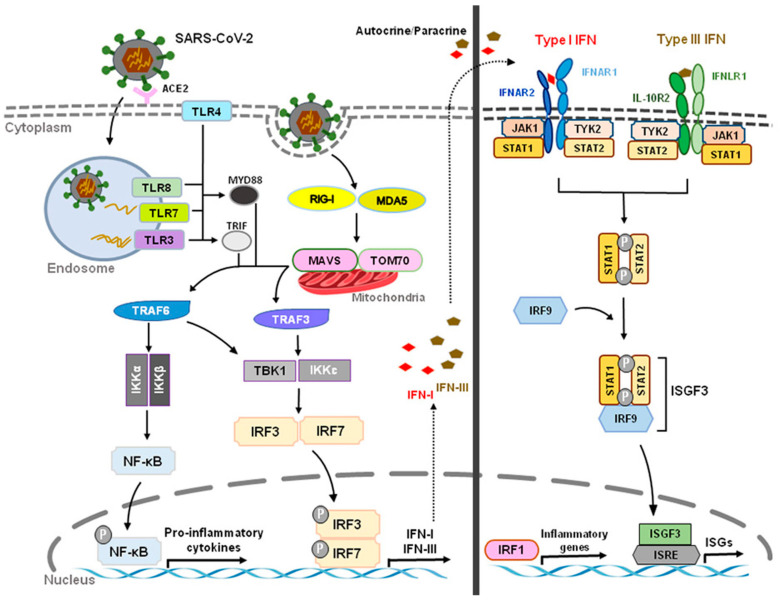
Innate Recognition and Interferon Signaling by Coronaviruses. Upon binding to angiotensin-converting enzyme 2 (ACE2) and subsequent sensing of coronaviruses by various pathogen recognition receptors, including toll-like receptors (TLRs) (TLR3, TLR4, TLR7, TLR8) and RIG-I-like receptors (RLRs) (RIG-I, MDA5), activation of transcription factors nuclear factor-kB (NF-kB) and interferon regulatory factor 3 and 7 (IRF3, IRF7) stimulates the production of pro-inflammatory cytokines and type I and III interferons (IFNs), respectively. The JAK/STAT signaling pathway is activated by IFNs through autocrine and paracrine secretion to induce the expression of IFN-stimulated genes (ISGs). Type I IFNs and IFN III IFNs are both capable of inducing ISGs, but type I IFN signaling produces a stronger and faster response as well as inducing pro-inflammatory cytokines and chemokines. However, delayed IFN-I responses not only fail to control SARS-CoV-2 virus but can also cause chronic inflammation and tissue damage.

**Figure 6:**
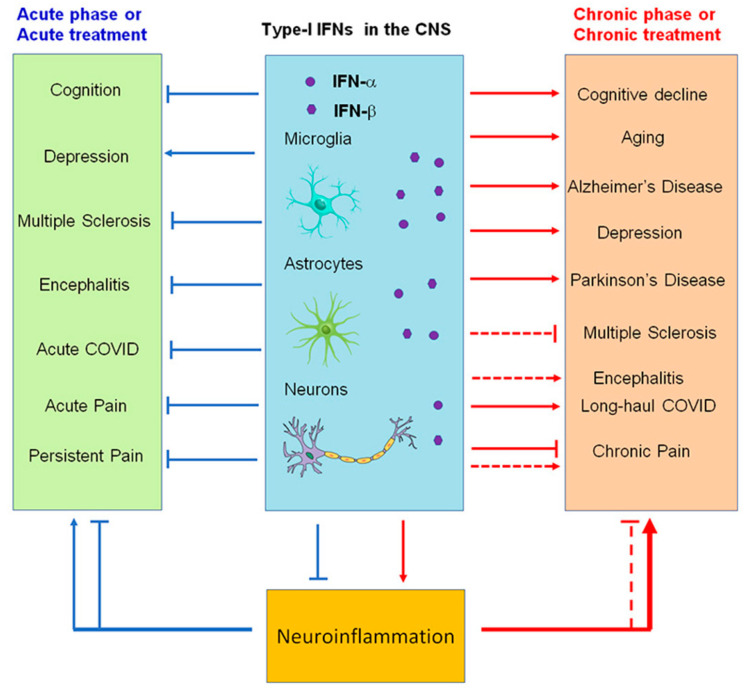
Modulation of neuroinflammation by type I IFNs in acute (blue) and chronic (red) neurological disease conditions. Note that IFN-α and IFN-β produce both beneficial and detrimental actions and may contribute to cognitive deficits (brain fog) in long-haul COVID.

Acute respiratory syndrome coronavirus 2 (SARS-CoV-2) that started in Wuhan, China, at the end of 2019 has become a global pandemic, termed COVID-19. Both SARS-CoV-2 and SARS-CoV enter human host cells via the angiotensin-converting enzyme 2 (ACE2) receptor. Upon cell entry, sensing of coronaviruses by various pathogen recognition receptors, including TLRs and RLRs, can trigger innate immune responses via activation of the transcription factors nuclear factor-kB (NF-kB) and interferon regulatory factor 3 and 7 (IRF3, IRF7), which will stimulate the production of pro-inflammatory cytokines and IFN-I and type III IFNs (Figure 5). These interferons are translated and secreted from the infected cells in an autocrine or paracrine manner. Upon binding type I IFN to the IFNAR1/IFNAR2 receptor and type III IFN to the IFNLR1/IL-10R2 receptor, signal transduction is initiated, resulting in the formation of the ISGF3 complex (IRF9/p-STAT1/p-STAT2). This complex then induces ISG expression. Through a variety of mechanisms, ISGs suppress viral replication by preventing viral entry and viral trafficking into the cell nucleus, inhibiting transcription/translation and degrading viral nucleic acids and blocking viral particle assembly. Compared to type III IFNs, IFN-I signaling induces a stronger and faster ISG response and may confer protection against acute virus infection [102]. (Figure 5 and Figure 6). 

As a result of acute COVID-19 infection, some patients develop physical and psychological symptoms. In this context, the term long-haul COVID (LC) is used to refer to symptoms lasting longer than 12 weeks, also called long COVID, chronic COVID syndrome, or post-acute sequelae of COVID-19 [103]. COVID-19 can cause LC in 10% to 30% of community-managed cases 2 to 3 months after infection [104,105] and can persist for over 8 months [106]. A number of LC symptoms can be seen, including severe relapsing fatigue, brain fog, coughing, chest tightness, and headache [107]. In addition to fatigue and poor concentration, post-COVID-19 syndrome is often accompanied by neuropsychiatric disorders, including sleep abnormalities, chronic headaches, “brain fog”, memory impairment, mood impairment, and pain syndromes as well as cardiac (palpitations, syncope, dysrhythmias, and postural symptoms) and respiratory (dyspnea and cough) symptoms [54,108]. A variety of hypotheses exist to explain the persistence of LC symptoms. Persistent elevation in the levels of type I (IFN-β) and type III (IFNλ1) interferon 8 months post infection were noted in patients with LC [109]. In this study, changes in IFNβ, pentraxin 3, IFNγ, IFNλ2/3, and IL-6 were noted as being able to predict LC with a range of 78.5% to 81.6% accuracy [109]. The association between these analytes and acute severe disease suggests that inflammation resolves slowly or defectively in LC individuals. Initial diminished IFN-I responses and enhanced IL-6 and tumor necrosis factor (TNF) responses were reported to be associated with the development of severe acute COVID-19 [110]. IFN-β was 7.92-fold higher in the LC group compared to the prevalent human coronaviruses group. After infection, IFN type I and III levels remained elevated for 8 months, in line with prolonged activation of pDCs, indicating chronic inflammation [109]. Early IFN-β treatment also protected against viral peak, while late treatment caused inflammation and life-threatening pneumonia [111]. The delayed IFN-I response, in contrast, manifests increased inflammation and tissue damage in addition to failing to control the virus. (Figure 6)

After the virus has been eliminated, the immune system develops a damaging, self-sustaining autoimmune response against self-tissue antigens, which is another immune dysregulation response that may contribute to the persistence of post-COVID symptoms. The presence of autoantibodies in patients with COVID have been demonstrated by several reports [112,113,114]. A number of autoimmune diseases and syndromes have been associated with COVID, such as idiopathic thrombocytopenic purpura [115] and Guillain–Barré syndrome [116]. Based on the cytoplasmic tyrosine-based motif associated with the receptor, Fcγ receptors could activate (Fcγ receptors I, III, and IV) or inhibit (Fcg receptor IIb) cells after binding by immunoglobulin G (IgG) antibody. There is evidence from recent studies that nociceptors express Fcγ receptor I activated by IgG after binding antigen and forming an antibody-antigen immune complex [117,118,119]. For example, in rheumatoid arthritis, the pain-related behavior induced by cartilage antibodies does not depend on inflammation in the joint but rather on recognition of tissue antigens, formation of local immune complexes, and activation of neuronally expressed Fcγ receptors. Many reports [117,118,119,120] have indicated a functional coupling between autoantibodies and pain transmission. Therefore, research into the novel contributions of autoantibodies to persistent pain may lead to new treatment strategies for both RA-related pain and pain resulting from other autoantibody-producing diseases, such as Guillain–Barré syndrome, systemic lupus erythematosus, and Sjogren’s syndrome.

In addition, PD [121] and cognitive impairments ranging from brain fog to AD acceleration [121,122] are among the neurodegenerative disorders described in patients with acute COVID. Many psychiatric conditions are also reported in post-COVID syndrome patients, including depression, anxiety, and post-traumatic stress disorder (PTSD) [123]. A COVID infection reportedly worsens PD’s clinical course by worsening motor and non-motor symptoms and causing anxiety with serious negative effects on mental health and quality of life. In AD patients, “cytokine storms” caused by inflammation of proinflammatory cytokines, such as interleukin-1 (IL-1) and IL-6, may synergize with amyloid-stimulated IFN-I levels, resulting in the “perfect storm” [124]. Viral particles and pathogens can be trapped within amyloid fibrils, which then trigger the “microglial neurodegenerative phenotype” [124]. In this subset of microglia, IFN-expressing cells are more prominent as a crucial component of AD and COVID infection that promotes complement cascade activation, synapse elimination, and immune activation [40]. Thus, some researchers hypothesized that affected patients may be at higher risk of developing cognitive decline after cure of COVID infection [125]. There is a possibility that this may result from directly adverse effects of the immune response, from accelerated or exacerbating pre-existing cognitive deficits, or from causing de novo neurodegenerative diseases. For controlling excessive immune responses, suppressing IFN responses could be a potential approach in AD and COVID [124]. The underlying pathophysiology of LC is poorly understood and needs further investigation.

## 5. Concluding Remarks and Future Perspectives 

Cells in the CNS produce IFN-I molecules to protect against viral infections. As an additional benefit, IFN-I s are currently used to treat inflammatory and autoimmune disorders as well as cancers, such as relapsing-remitting multiple sclerosis [126,127], cancer [128], and chronic hepatitis C [129]. However, CNS cells may also produce excessive amount of IFN-I s, which can cause autoimmune disease, chronic and congenital infection, and genetic disorders [57,130], generally called “cerebral interferonopathies”. Furthermore, both mice and patients [51,52] have been reported to display the symptoms of cognitive impairment, anxiety, depression, and delirium after being exposed to IFN-Is for a prolonged period of time. Although the IFN-Is are involved in CNS inflammation and neurological disease, they can produce either protective or detrimental effects on the disease depending on the disease. As stated in above section, increased expression of IFN-I has been found in the brains of patients with AD and was proven to contribute to the formation of disease in an AD model of mouse [86]. However, some studies reported conflicting results that showed a neuroprotective effect of IFN-Is in human and murine models with AD-like neurodegeneration and PD-like dementia [32,131]. Findings in mouse models for the autoimmune disease MS suggested that IFN-Is have a protective effect in mice. The disease will be exacerbated in the absence of IFN-I signaling [89]. By contrast, IFN-I signaling is required for formation of neuromyelitis optica of another autoimmune CNS disease [63,132]. Increased expression of IFN-I in the brain is a characteristic of physiological aging in humans and mice [80]. In vivo experiment showed that blockade of IFN-I signaling could improve cognitive function in aged mice [80]. 

In pain research, both beneficial and detrimental effects of IFN-I have been demonstrated [64,65,66,68,69,70,94]. However, it is still largely unknown what extent of beneficial and detrimental effects of IFN-I are mediated by astrocyte, microglia, neurons, and other cells in the CNS (Figure 6). Therefore, much work is still required to clarify their roles in specific neurological diseases. It appears that timing of IFN-I induction relative to viral replication determines the response outcome, with earlier IFN-I induction or administration triggering a more favorable response. In contrast, a delayed IFN-I response leads to inflammation and tissue damage as well as failure to control the virus. It may therefore be beneficial to supplement IFN-I early in the disease course, especially when IFN-I expression is compromised because of viral suppression or aging. For potential therapeutic applications with IFN-Is, it will be essential to identify cellular targets that can limit or reverse the effects of IFN-I-associated inflammation. Several mechanisms may be involved, including inhibiting inflammatory genes downstream of the IFN-I signal as well as promoting the negative feedback mechanism in response to IFNs. New insights into modulations of neuroinflammation by IFNs may lead to new therapeutics for the control of neurological diseases, including cognitive decline, chronic pain, and long-haul COVID.

## Figures and Tables

**Table 1:** Roles of IFN-α and IFN-β in neurological diseases. IFN-α and IFN-β produce both beneficial and detrimental actions and may contribute to cognitive deficits (brain fog) in long-haul COVID.

Pathological Conditions	Species	Effects	Mechanisms	References
Encephalitis, IFN-α/β	Mouse	Beneficial	Increases microglia and myeloid cell activation	[37,47]
Depression, IFN-α	Human	Detrimental	Reduces serotonin production	[48,49]
Depression, IFN-α	Rhesus Monkey	Detrimental	Reduces neurogenesis in the hippocampus	[50]
Dementia/aging, IFN-β	Human	Detrimental	Causes memory impairment by decreasing hippocampus neurogenesis	[51,52]
Dementia/aging, IFN-β	Mouse	Detrimental	Disrupts endogenous neurogenesis in the hippocampus	[53]
Infection/brain fog, IFN-β	Human	Detrimental	Induces “brain fog” by promoting neuroinflammation	[54]
Alzheimer’s disease, IFN-β	Mouse	Detrimental	Induces memory impairment by decreasing hippocampus neurogenesis	[40,55]
Alzheimer’s disease, IFN-α	Human	N/A	Upregulation in microglial cells	[56]
Alzheimer’s disease, IFN-β	Human	Beneficial	Shows some beneficial effects in cognitive subscale in early AD patients	[57]
Parkinson’s disease, IFN-α	Human	Detrimental	Increases neuroinflammation in the striatum	[58]
	Mouse	Detrimental	Induces dopaminergic cell death in the striatum	[59,60]
Parkinson’s disease, IFN-β	Mouse	Beneficial	Loss of IFNAR forms Lewy body and causes PD-like dementia	[32]
Multiple sclerosis, IFN-β	Mouse	Beneficial	Inhibits production of myelin-damaging products	[61,62]
Multiple sclerosis, IFNAR	Mouse	Detrimental	EAE-induced demyelination is protected in IFNAR-knockout mice	[63]
Multiple sclerosis, IFN-β	Mouse	Beneficial	Decreases serum levels of cytokines	[25]
Naïve condition, intracranial IFN-α	Rat	Antinociception	Mu-opioid-receptor-dependent	[64]
Naïve/CFA model, intrathecal IFN-α	Rat	Antinociception	Opioid-receptor-dependent	[65]
Naïve/CFA model, intrathecal IFN-α	Rat	Antinociception	Inhibits EPSC and capsaicin-induced P-ERK	[66]
LPS model, intrathecal IFN-β	Mouse	Antinociception	IFNAR1-mediated and TLR-mediated actions	[67]
Spared nerve injury model, intrathecal IFN-β	Mouse	Antinociception	Induction of ISG-15 and inhibition of MAPK	[68]
Arthritis model, intrathecal IFN-β	Mouse	Combined injection of TNF antibody produces antinociception	Induction of IL-10 expression	[69]
Naïve condition, intraplantar IFN-α, IFN-β	Mouse	Hyperalgesia	Activation of MAPK and MNK-elF4e translation	[70]
Naïve condition, intrathecal IFN-α, IFN-β	Mouse	Antinociception	IFN-I–IFNAR1 signaling; inhibition of Nav1.7 and calcium channel activities	[5]
